# 111. Assessment of Empiric Management Practices of Common Infections in Solid Organ Transplant Recipients at a Canadian Tertiary Care Centre - A Retrospective Cohort Study

**DOI:** 10.1093/ofid/ofab466.313

**Published:** 2021-12-04

**Authors:** Syed Z Ahmad, Sagar Kothari, Michelle Zhao, Abbigayle Teixeira-Barreira, Mark Richmond, Shahid Husain

**Affiliations:** 1 Toronto General Hospital Research Institute, University Health Network, Oakville, Ontario, Canada; 2 University Health Network, University of Toronto, Toronto, ON, Canada

## Abstract

**Background:**

Post-transplant infections remain a leading cause of morbidity and mortality in solid organ transplant recipients (SOTR). Standardized antimicrobial treatment guidelines for infectious syndromes may contribute to improved clinical care. Our study seeks to assess the rate of therapeutic compliance with local standard guidelines in the treatment of common infections in SOTR, and their associated outcomes.

**Methods:**

Consecutive adult SOTR admitted to the transplant floor from January–May 2020 for treatment of an infectious syndrome of interest were reviewed for study inclusion. Patients were followed until discharge or for 30 days following the date of diagnosis, whichever was shorter. Data was extracted from electronic medical records.

**Results:**

475 SOTR were admitted to the transplant floor, of which 156 patients (33%) were admitted with infectious syndromes. Guidelines were applicable to 117 patients, constituting the following 122 syndromes: 51 pneumonias; 34 urinary tract infections (UTI); 22 bacteremias and 15 intra-abdominal infections (Fig. 1). Intra-abdominal infections occurred earliest at a median time of 9 months post-transplant followed by bacteremias, pneumonias, and UTIs (medians 10, 38 and 54 months respectively) (Table 1). 47% of patients were empirically treated with a regimen compliant with guidelines and 66% were provided compliant tailored therapies. Non-compliance with empiric management guidelines resulted in a significantly higher proportion of patients requiring ICU transfer when compared to compliance (25% vs. 9%; *P* = .02) (Table 2). Non-compliance with tailoring protocols resulted in an increased overall length of stay (medians 11 days vs. 8 days; *P* = .04). Within 30 days of discharge, no differences in readmission, development of *Clostridium difficil*e infection, rejection, graft loss or death were observed between patients receiving compliant or non-compliant regimens.

Figure 1. Study Flow Diagram

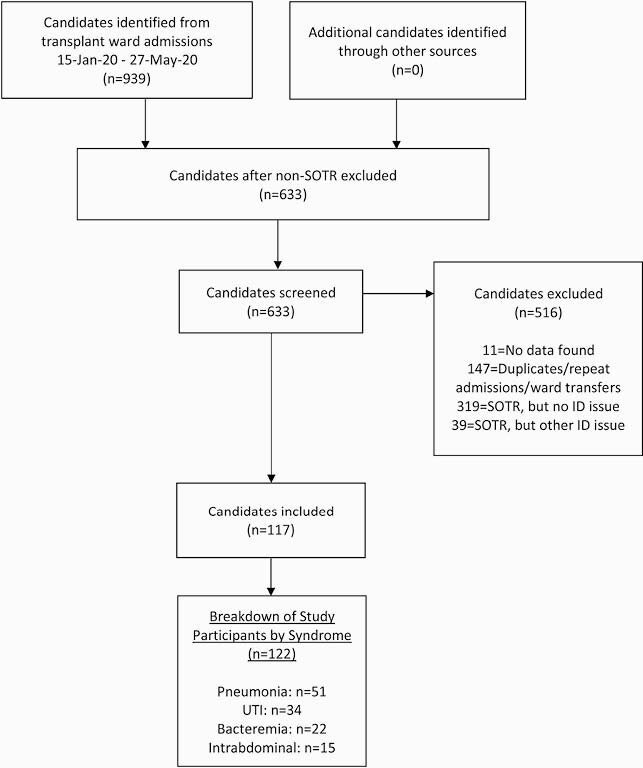

Table 1. Baseline Characteristics of Patient Cohort

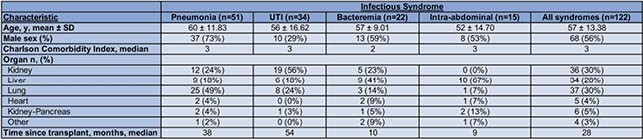

Table 2. Outcomes of compliant vs. non-compliant treatment in patients receiving antimicrobial therapy for an infectious syndrome

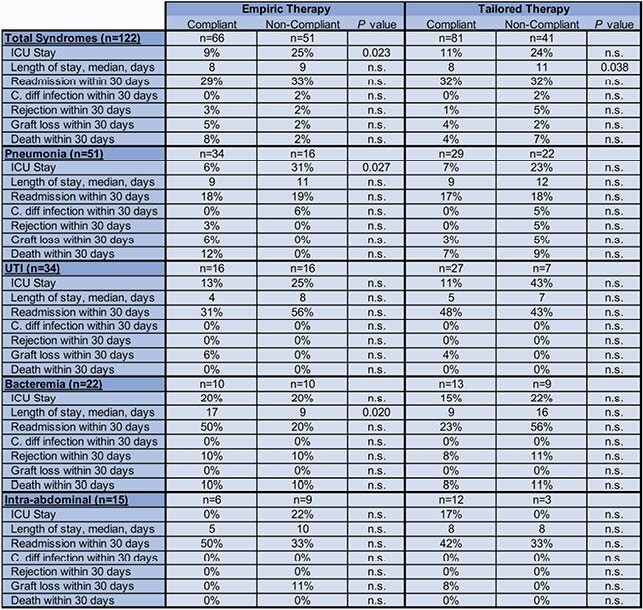

**Conclusion:**

Non-compliance with locally developed antimicrobial management guidelines resulted in a higher proportion of patients being transferred to the ICU and an increased length of stay in our cohort, highlighting the benefits of adherence. Future studies will assess long-term outcomes associated with compliance to infection management guidelines.

**Disclosures:**

**All Authors**: No reported disclosures

